# Gene expression differences in differentially methylated sites associated with HIV status and cocaine use

**DOI:** 10.1002/ctm2.70466

**Published:** 2025-09-19

**Authors:** Eric J. Earley, Bryan C. Quach, Fang Fang, Laura J. Bierut, M‐J S. Milloy, Kanna Hayashi, Kora DeBeck, Dana B. Hancock, Bradley E. Aouizerat, Ke Xu, Eric Otto Johnson

**Affiliations:** ^1^ GenOmics and Translational Research Center RTI International Durham North Carolina USA; ^2^ Department of Psychiatry Washington University School of Medicine St. Louis Missouri USA; ^3^ British Columbia Centre on Substance Use Vancouver British Columbia Canada; ^4^ Department of Medicine University of British Columbia Vancouver British Columbia Canada; ^5^ Faculty of Health Sciences Simon Fraser University Burnaby British Columbia Canada; ^6^ School of Public Policy Simon Fraser University Vancouver British Columbia Canada; ^7^ Bluestone Center for Clinical Research, College of Dentistry, New York University New York New York USA; ^8^ Department of Oral and Maxillofacial Surgery College of Dentistry, New York University New York New York USA; ^9^ Department of Psychiatry School of Medicine, Yale University New Haven Connecticut USA; ^10^ VA Connecticut Healthcare System West Haven Connecticut USA; ^11^ RTI Fellow Program RTI International Durham North Carolina USA

1

Dear Editor,

Advances in antiretroviral therapy (ART) have made HIV a chronic, manageable disease for people living with HIV (PLWH) with consistent ART access. However, HIV still increases risk of non‐AIDS defining conditions such as cancer and impaired cognitive function.^1^ DNA methylation and gene expression dynamics appear to play a role in these comorbid disease‐related processes.^2^ Furthering a mechanistic understanding of the risk of non‐AIDS defining conditions, this study is the first to demonstrate that many immune response genes, previously identified via epigenome‐wide association studies (EWAS) of DNA methylation, also show consistently upregulated gene expression in PLWH, especially among those with detectable viral load despite ART and those who recently used cocaine.

HIV‐1 infection is characterised by persistent immune activation and chronic inflammation.^3^ DNA methylation – particularly at CpG sites near immune‐regulatory genes – has been linked to HIV acquisition, viral control, and comorbidities such as cancer and impaired cognitive function.^1^ Recent EWAS have identified hypomethylation in key antiviral response genes such as *MX1* and *NLRC5*, and hypermethylation in genes linked to HIV progression including *CX3CR1* and *TNF*.^1^ However, it remains unclear whether such methylation changes translate into changes in gene expression. Moreover, cocaine use, common among PLWH and associated with faster disease progression, may further influence gene expression via epigenetic mechanisms.^4^

To investigate this, we analysed gene expression via RNAseq in whole blood from 588 individuals enrolled in the Vancouver People Who Inject Drugs Study (VPWIDS), including 227 PLWH (38.6%) with a mean age of 49.6 years (± 9.6 SD; Table 1).^5^ All PLWH were on ART at the time of blood draw, 194 had undetectable viral load (< 200 viral copies/mL), and 33 had detectable viral load (mean 18 850 viral copies/mL). The cohort had high prevalence of cocaine, opioid, and methamphetamine use. Focusing on cocaine use (crack and powder), 121 PLWH (47%) and 180 HIV‐negative (50%) participants reported recent use.

**TABLE 1:** Vancouver people who inject drugs study characteristics.

	PLWH with undetectable viral load *N* = 194	PLWH with detectable viral load *N* = 33	HIV‐negative *N* = 361	*p* ^*^
Age, year (mean ± SD)	51.1 ± 8.4	48.5 ± 8.4	48.9 ± 10.2	.4
Sex, no. (male, %)	148 (76.3)	26 (78.8)	265 (73.4)	1.0
Genetic inferred ancestry, no. (EU, %)	194 (100)	33 (100)	361 (100)	–
Viral load, copies/mL (mean ± SD)	37.8 ± 1.3	5992.1 ± 8.7	0 ± 0	–
Cocaine, no. (%)	58 (29.9)	9 (27.3)	95 (26.3)	1.0
Crack, no. (%)	82 (42.3)	10 (30.3)	137 (38.0)	1.0
Any cocaine, no. (%)	107 (55.2)	14 (42.4)	180 (49.9)	1.0
Heroin, no. (%)	69 (35.6)	14 (42.4)	177 (49.0)	.03
Opioid, no. (%)	86 (44.3)	15 (45.5)	195 (54.0)	.3
Meth., no. (%)	75 (38.7)	13 (39.4)	115 (31.9)	.9
Cannabis, no. (%)	30 (15.5)	4 (12.1)	34 (9.4)	.5
Any stimulant, no. (%)	151 (77.8)	22 (66.7)	242 (67.0)	.2
Any non‐prescript, no. (%)	170 (87.6)	28 (84.8)	292 (80.9)	.5

Abbreviations: EU, European; PLWH, people living with HIV; SD, standard deviation.

* Univariate tests were performed using the full PLWH cohort (*N* = 227); *p*‐values were adjusted using Bonferroni method.

A PubMed search using ‘HIV’ and ‘epigenome’ identified five in vivo human whole blood–based EWAS relevant to HIV acquisition or severity (Table S1). From these, we selected 18 genes for expression analyses based on stringent criteria: (1) harbouring CpG sites differentially methylated in association with HIV acquisition or severity that were independently replicated in at least one other study, or (2) containing CpG sites identified as significant mediators of cocaine's impact on HIV severity in prior mediation analyses. Expression differences by HIV status were assessed using negative binomial regression, adjusting for sex, age, RNA Integrity Number (RIN), the top 5 genotype PCs, and proportions of 5 immune cell classes (Supplemental Methods).

We observed significant upregulation of 9 out of the 18 target genes in PLWH versus HIV‐negative participants: *EPSTI1, IFI44L, IFIT3, MX1, NLRC5, PARP9, PLSCR1, RIN2*, and *RSAD2* (Table 2, Figure 1), after Bonferroni correction. These genes were previously reported with hypo‐methylated CpG sites in PLWH.^3^, ^5^, ^6^ Sensitivity analysis stratified by viral load confirmed upregulation in both virally suppressed (*N* = 194) and viremic PLWH (*N* = 33), with two additional genes (*TAP1* and *TNIP3*) differentially expressed among the latter (Table S2).

**TABLE 2:** Differential gene expression results for target genes.

Gene	Methylation^a^	Published CpG	Gene location^b^	Base mean	LFC	*p* ^c^
*IFI44L*	Hypo^6^	cg13452062, cg05696877	5’UTR, 5’UTR	3473.3	.877	**2.38 × 10^−^** **^7^**
*EPSTI1*	Hypo^2^	cg03753191	TSS1500	2975.5	.549	**2.68 × 10^−6^**
*RIN2*	Hypo^2^	cg26396492	Gene body	326.6	.210	**1.15 × 10^−5^**
*PLSCR1*	Hypo^2^	cg06981309	5’UTR	1522.8	.335	**4.84 × 10^−5^**
*RSAD2*	Hypo^6^	cg10771443, cg15839328	Gene body, Gene body	2943.1	.668	**8.70 × 10^−5^**
*MX1*	Hypo^2^, ^4^	cg26312951	TSS200	10207.9	.428	**6.39 × 10^−4^**
*PARP9*	Hypo^2^	cg22930808, cg08122652	5'UTR, 5’UTR	6036.1	.206	**9.63 × 10^−4^**
*NLRC5*	Hypo^1^, ^2^, ^3^, ^4^, ^5^, ^6^	cg16411857, cg07839457, cg05757530	TSS1500, TSS1500, 5′UTR	8825.3	.115	**2.53 × 10^−3^**
*IFIT3*	Hypo^2^	cg06188083	Gene body	9823.4	.325	**6.16 × 10^−3^**
*TNIP3*	Hyper^1^	cg26832294, cg10061361	Gene body, Gene body	23.6	.141	.14
*TNF*	Hyper^6^	cg10717214, cg21222743, cg04425624	1stExon, 1stExon, 1stExon	273.5	–.080	.16
*TAP1*	Hypo^2^	cg08818207	Gene body	12385.7	.081	.26
*CX3CR1*	Hyper^2^, ^4^	cg22917487	TSS200/body	6085.7	–.070	.80
*CD44*	Hypo^1^	cg20971158	TSS1500	13507.4	.035	1.00
*HCP5*	Hypo^1^	cg18808777, cg25843003, cg21684411	3'UTR, 3'UTR, 3'UTR	3792.2	–.026	1.00
*IFITM1*	Hypo^2^	cg03038262	3'UTR	27092.0	.056	1.00
*RASSF3*	Hyper^6^	cg18544413	Gene body	4961.5	.016	1.00
*TAP2*	Hypo^2^	cg22940798	Gene body	4992.5	.068	1.00

^a^ Methylation status from literature: (1) Zhang, et al. (2023); (2) Shu, Justice, et al. (2020); (3) Shiau et al. (2019); (4) Zhang et al. (2017); (5) Zhang et al. (2016); (6) Shu, Jaffe et al. (2020). LFC – log2 fold change where positive values reflect higher gene expression in PLWH compared to HIV‐negative.

^b^ Gene location indicates the genomic context of the associated CpG site: TSS200 (≤200 bp upstream of TSS), TSS1500 (≤1500 bp upstream of TSS), 5′UTR, 1stExon, Gene body, 3′UTR. Multiple locations indicate the CpG maps to more than one region.

^c^ *p* Values were adjusted using Bonferroni method.

Bold values indicate statistical significance *p* < .05.

**FIGURE 1:**
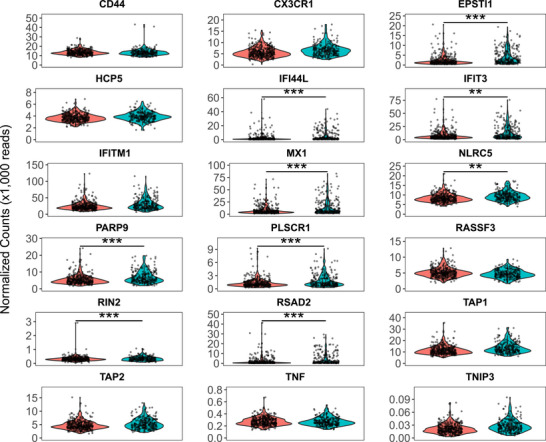
Differential gene expression of 18 targeted genes. Shown are violin plots of normalised mRNA read counts (gene‐level) based on HIV status, with one gene per panel. Red represents participants who were HIV‐negative, and blue/green represents PLWH. **p < .01; ***p < .001.

Transcript‐level analysis confirmed differential expression of isoforms for all nine genes and revealed additional expression changes in *CD44*, *RASSF3*, and *TAP1*. Specifically, three transcripts for *CD44* (*ENST00000442151*, *p < *.001; *ENST00000528922*, *p* = .01; *ENST00000531118, p* = .04), one transcript for *RASSF3* (ENST00000540088, *p *= .02) and two for *TAP1* (ENST00000487296, *p <* .001; ENST00000486332, *p *= .003) were upregulated in PLWH (Figure 2). Transcript‐level sensitivity analysis showed some isoforms of *CD44, IFIT3, NLRC5*, *PLSCR1*, and *TAP1* were significantly upregulated only in PLWH who had detectable viral load but not in those from PLWH who had undetectable viral load (Table S3), including two protein coding isoforms: *ENST00000371811* (*IFIT3)* and ENST00000462666 (*PLSCR1*). These findings suggest a nuanced transcriptional response linked to viral replication status. The selective upregulation of specific isoforms may reflect post‐transcriptional regulatory mechanisms such as alternative splicing, differential promoter usage, or isoform‐specific mRNA stability, which could fine‐tune protein function and immune signalling in response to viral replication (Figure S1).

**FIGURE 2:**
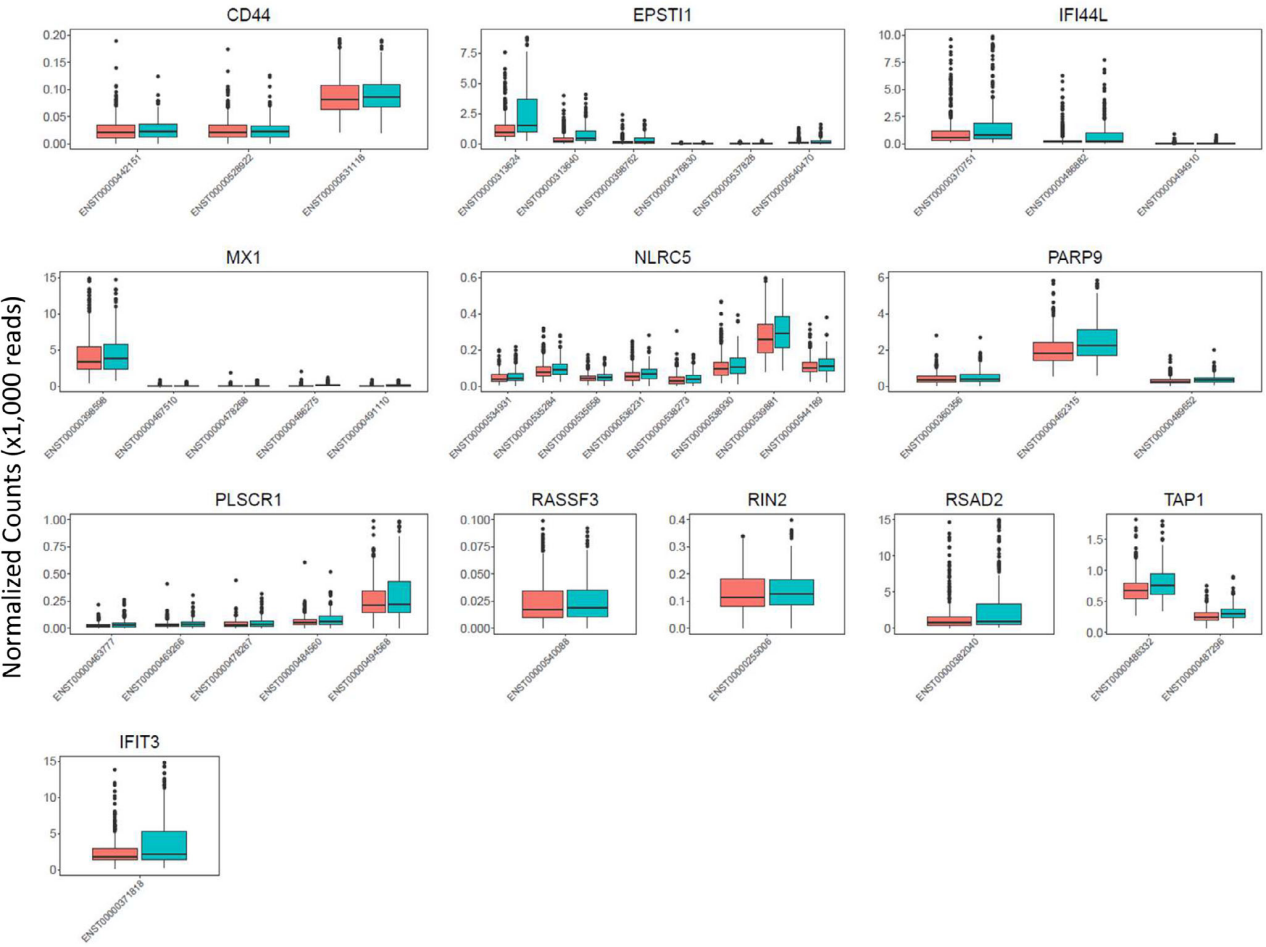
Differential transcript expression results. Shown are boxplots plots of normalised mRNA read counts (transcript‐level) based on HIV status with one gene per panel. Red represents participants who were HIV‐negative and blue/green represents PLWH. Differences in scale compared to Figure 1 reflect transcript‐level versus gene‐level quantification. Only transcripts exhibiting *p* < .01 are shown.

We next examined gene expression associated with HIV status stratified by cocaine use (Supplemental Methods; Table S4). Two comparisons were made: (1) PLWH with recent cocaine use (*N* = 121) versus HIV‐negative with recent use (*N* = 180), and (2) PLWH with no recent use (*N* = 106) versus HIV‐negative with no recent use (*N* = 181). Among PLWH with recent cocaine use, *IFI44L, RIN2, PLSCR1, MX1*, and *RSAD2* were significantly upregulated (Table S5), while *EPSTI1* upregulation was observed only in non‐recent users. Transcript‐level upregulation specific to PLWH using cocaine was noted for isoforms of *PARP9, MX1, PLSCR1*, and *NLRC5*, but not in PLWH without cocaine use (Table S6). Prior work in an independent study showed methylation changes mediating the association between cocaine use and HIV severity,^4^ and the current study support this model by confirming concordant gene expression changes.

Pathway analysis of the 12 differentially expressed genes (Supplemental Methods) – including the nine genes significantly upregulated (*EPSTI1, IFI44L, IFIT3, MX1, NLRC5, PARP9, PLSCR1, RIN2*, and *RSAD2*) and three genes upregulated only in specific isoforms (*CD44, RASSF3*, and *TAP1*) – revealed enrichment for the interferon alpha/beta signalling pathway (*p *= 1.75 × 10^−6^, FDR), as well as its parent pathways, including interferon signalling (*p = *1.83 × 10^−4^, FDR) and Cytokine Signalling in Immune system (*p = *0.02, FDR; Table S7). To place these findings in a broader transcriptional context, we next examined whether other genes in the enriched pathways were also dysregulated, even if not directly linked to DNAm in our dataset. Among 80 genes upregulated in response to interferon‐alpha,^7^ 33 were significantly differentially expressed in this study (*p* < .05; Table S8). *NLRC5*, one of our top DNAm‐ and expression‐associated genes, is a key regulator of both MHC class I and NF‐kB activation of pro‐inflammatory genes.^8^, ^9^ Although MHC class I genes were not differentially expressed, 32 of 200 known NF‐kB targets were dysregulated in PLWH, highlighting the pathway's role in HIV‐associated immune activation (Table S8). These findings suggest that, in HIV, chronic immune activation may shift *NLRC5* activity toward NF‐kB regulation, potentially driven by interferon feedback or viral immune evasion.

Lastly, a drug repurposing screen (Supplemental Methods) using the 12 target genes identified a single potential therapeutic candidate: *bivatuzumab*, a monoclonal antibody targeting *CD44* (Table S9). This prodrug targets three isoforms of *CD44*, including one protein‐coding isoform ENST00000442151, which was significantly upregulated in the current study. CD44 is involved in lymphocyte activation and migration, suggesting potential in inflammatory disease treatment.^10^

In conclusion, this study links methylation patterns to gene expression changes in innate immune genes among PLWH. Our findings support a model where HIV and cocaine use contribute to transcriptional dysregulation through epigenetic mechanisms, particularly in interferon‐responsive pathways. These insights may guide biomarker development and therapeutic interventions.

## AUTHOR CONTRIBUTIONS

BEA, KX and EOJ contributed to the overall study design; EJE, BCQ and FF conducted statistical analyses; EJE and FF drafted the article; and all authors interpreted the results and contributed to the final version of the article.

## CONFLICT OF INTEREST STATEMENT

The authors declare no conflicts of interest.

## ETHICS STATEMENT

The VIDUS, ACCESS, and VPWIDS studies received review and approval from the University of British Columbia/Providence Healthcare Research Ethics Board. All participants were aged 18 years and older, and provided written informed consent prior to their involvement in the studies.

## FUNDING INFORMATION

This project was supported by the National Institute on Drug Abuse through the following grants: R01DA038632 (Johnson), R61DA047011 (Johnson and Aouizerat), R33DA047011 (Johnson and Aouizerat), R01DA051908 (Johnson and Jacobson), U01DA038886 (Hayashi and DeBeck), and U01DA021525 (Milloy).

## Supporting information



Supporting Information

Supporting Information
