# Supplementary material for: Gene expression differences in differentially methylated sites associated with HIV status and cocaine use
Source: Clin Transl Med. 2025 Sep 19;15(9):e70466. doi: 10.1002/ctm2.70466 (PMC12446875; doi:10.1002/ctm2.70466)
Supplement: Supplementary file 1 — Supporting Information [file CTM2-15-e70466-s002.docx]

# **Methods S1**

*Cohort description*

Details of the VPWIDS have been previously described^1^. Briefly, VPWIDS participants originated from either new community recruitment or selection from existing cohort studies. For the new recruitment, participants were recruited from Vancouver’s Downtown Eastside neighborhood, an area with widespread illicit drug use and HIV infection. Participants were ≥ 18 years old, used any illicit drug via injection at least once in the 30 days prior to enrollment, and provided written informed consent. Recruited participants completed an interviewer-administered questionnaire to provide harmonious data with existing studies on socio-demographics, substance use patterns, and sexual behaviors; underwent a test of HIV serostatus; and if HIV seropositive, plasma HIV RNA-1 viral load and viral genotyping.

For the selection from existing cohort studies, VPWIDS participants were drawn from either (1) the AIDS Care Cohort to evaluate Exposure to Survival Services (ACCESS), a prospective cohort of people living with HIV infection who use illicit drugs; or (2) the Vancouver Injection Drug Users Study (VIDUS), a prospective cohort of people at risk of HIV infection who use illicit drugs. VIDUS/ACCESS participants completed an interviewer-administered questionnaire on socio-demographics, HIV risk behaviors, HIV treatment patterns, substance use patterns, social/structural exposures, and other relevant exposures/outcomes.

The VIDUS, ACCESS and VPWIDS studies were reviewed and approved by the University of British Columbia/Providence Healthcare research ethics board. All VPWIDS participants provided blood specimens for extraction of human DNA and mRNA extraction. All participants were of European descent. Recent cocaine use was defined as reporting cocaine use at least once in six months before the baseline interview. Viral suppression was defined as <200 viral copies/mL of whole blood. ^2^

*Sex as a biological variable*

Our study enrolled self-reported male and female participants, although we note the majority of participants were male. To reduce the risk of accidental mis-labeling of bio-samples during DNA and RNA sequencing, discrepancies between self-reported sex and sex defined by chromosome Y gene expression resulted in exclusion of that participant in the study. Four samples (two self-reported males and two self-reported females) were removed due to such discrepancies.

Sex was included as a covariate in all statistical models; however, the small number of female participants limited the statistical power for sex-stratified analyses. Given evidence for sex-based differences in immune function and HIV pathogenesis, future studies with larger, more balanced cohorts are needed to determine whether the observed associations differ by sex.

*Deriving target genes from HIV-associated CpGs*

A literature search for EWAS within HIV acquisition, severity, or both, was performed using PubMed by searching for keyword terms “HIV” and “epigenome.” To ensure cross-study comparability, citations were excluded unless data were derived *in vivo* from human whole blood. In addition, only epigenome-wide studies were considered. Studies focused on interrogating the epigenetic clock were excluded. Five publications were selected by this process, and from these 18 CpG sites were selected. These CpG sites showed epigenome-wide significant differential methylation either associated with HIV acquisition and independently replicated, or as significant mediators of the effect of cocaine use on HIV severity. A list of proximal protein-coding genes to these CpG sites was identified, and these 18 genes were subject to differential gene and transcript expression analysis (**Supplemental Table 1**).

*RNA sequencing and DNA Genotyping*

RNAseq libraries were prepared from whole blood samples using the NuGEN Universal Plus mRNA-seq kit with human globin AnyDeplete on the Illumina Nextseq platform by the Rutgers University Cell and DNA Repository (RUCDR Infinite Biologics). Trimmed reads were mapped to the GRCh38 human reference transcriptome (GENCODE v28) with HISAT2 v2.1.0.^3^ Transcript-level quantification of RNA was estimated with Salmon v0.11.2 ^4^ using GC bias correction, and gene-level quantifications were derived from these transcript quantification estimates using the R library, *tximport.*^5^ Additional quality control metrics were generated using FASTQC.^6^

VPWIDS participants’ DNA was genotyped on the Illumina Infinium OmniExpress-24 BeadChip. DNA variant calling was also performed on samples from the same study participants and concordance between RNA and DNA single nucleotide variants (SNVs) and small insertions-deletions (indels) was used to identify potentially mislabeled samples. RNA variants were called using the *mpileup* function within *SAMtools,* ^7^ and problematic sample pairs between genotyping array and RNAseq were identified using Pearson’s *r* correlation. If samples with conflicting IDs exhibited *r* >0.8 between DNA and RNA variant calling they were assumed to be incorrectly labeled and IDs were updated to match each other. If samples with matching IDs exhibited *r* >0.6 they were assumed to be correctly matched. Discrepancy between the *k*-means (*k*=2) clustering of sex assignments and the mean Y chromosome gene expression identified a further set of potentially problematic samples. As a final QC step, we excluded samples with low transcript diversity (lower quartile - 1.5*IQR), high read duplication (>75%), and missing genotype data. Overall, these filters removed 14 samples resulting in a final analysis set of 588 samples, 227 PLWH samples (i.e., samples from PLWH), and 361 HIV-negative samples.

*Cell type deconvolution*

To control observed gene expression bias due to variation in cell types across individuals, we performed cell type deconvolution using the tool CIBERSORTx ^8^ with the LM22 leukocyte cell-type reference.^9^ All 22 cell types were used to estimate per-sample cell type proportions. However, to replicate the approach of prior methylation studies,^10-14^ only 5 cell classes were considered in differential expression models – CD4+ T cells, CD8+ T cells, B cells, Granulocytes, and Monocytes. The higher resolution proportions of the 22 cell types were collapsed into these categories via summing the sub-types (e.g., resting Mast cells + activated Mast cells + Eosinophils + Neutrophils = Granulocytes) except for NK T cells which were not present in the LM22 reference.

*Statistical analysis*

Unless otherwise noted, all reported *P*-values were adjusted for multiple hypothesis testing using the conservative Bonferroni correction to reduce type 1 error rates. Cohort characteristics were tested for association with HIV status in univariate logistic regression. Differential expression analysis was performed with *DESeq2*,^15^ which fits negative binomial regression models for each gene/transcript with the gene/transcript expression as the outcome variable and HIV status as the primary independent variable. Age, sex, RNA quality (RIN), 5 deconvolution-derived cell class proportions, and the top 5 Principal Components calculated from observed and LD-pruned array genotypes were included as model covariates. To control for potential bias due to the higher variance of lowly expressed genes, we filtered out genes with mean depth <10 reads across 35% of samples and applied apeGLM shrinkage.^16^ Target genes were extracted from the transcriptome-wide results. Differential transcript usage was measured using the tool *DEXSeq^17^* in a multivariate linear model including the same covariates as the differential gene/transcript analysis. Unless otherwise noted all reported *P*-values are adjusted using the Bonferroni method. Gene sets for NF-kB (‘HALLMARK_TNFA_SIGNALING_VIA_NFKB’) and IFN-alpha activation (‘HALLMARK_INTERFERON_ALPHA_RESPONSE’) were taken from the MSigDB database^18^ for expression analysis of genes in molecular pathways related to the 18 target genes.

*Sensitivity analysis of viral load*

To assess the impact of viral load on differential gene and transcript expression, a stratified analysis was performed where the subset of participants who exhibited detectable viral load (>200 viral copies/mL of whole blood; N=33) was compared with HIV-negative participants (N=361). A parallel comparison was also made between PLWH exhibited no detectable viral load (N=194) and the HIV-negative participants (N=361).

*Stratified analysis of cocaine use*

To assess the impact of cocaine use on gene and transcript expression, a stratified analysis of cocaine use was performed. Participants were grouped based on their use of cocaine (injection cocaine or crack cocaine) in the six months prior to sample collection. PLWH who reported using cocaine, regardless of viral load status (N=121), were compared to HIV-negative participants who also reported using cocaine (N=180). A parallel comparison was then made between PLWH who reported no cocaine use (N=106) and HIV-negative participants who reported no cocaine use (N=181).

*Drug repurposing analysis*

To identify potential drug candidates, we used a drug repurposing analysis pipeline developed by our group.^19^ This approach integrates multiple drug repurposing databases with biological data to identify compounds that target genes associated with a specific condition. Our input consisted of genes that exhibited significant differential gene expression between PLWH and HIV-negative groups.

The analysis queried four drug databases: Target Central Resource Database, Open Targets, Therapeutic Target Database, and DrugBank. Drug annotations were collected included, including known gene targets, clinical status (approved, experimental, preclinical), and target selectivity (number of gene targets). All drugs with gene targets that overlapped with our 12 differentially expressed genes were collected for further analysis. A comprehensive cross-resource summary was then created to consolidate this information.


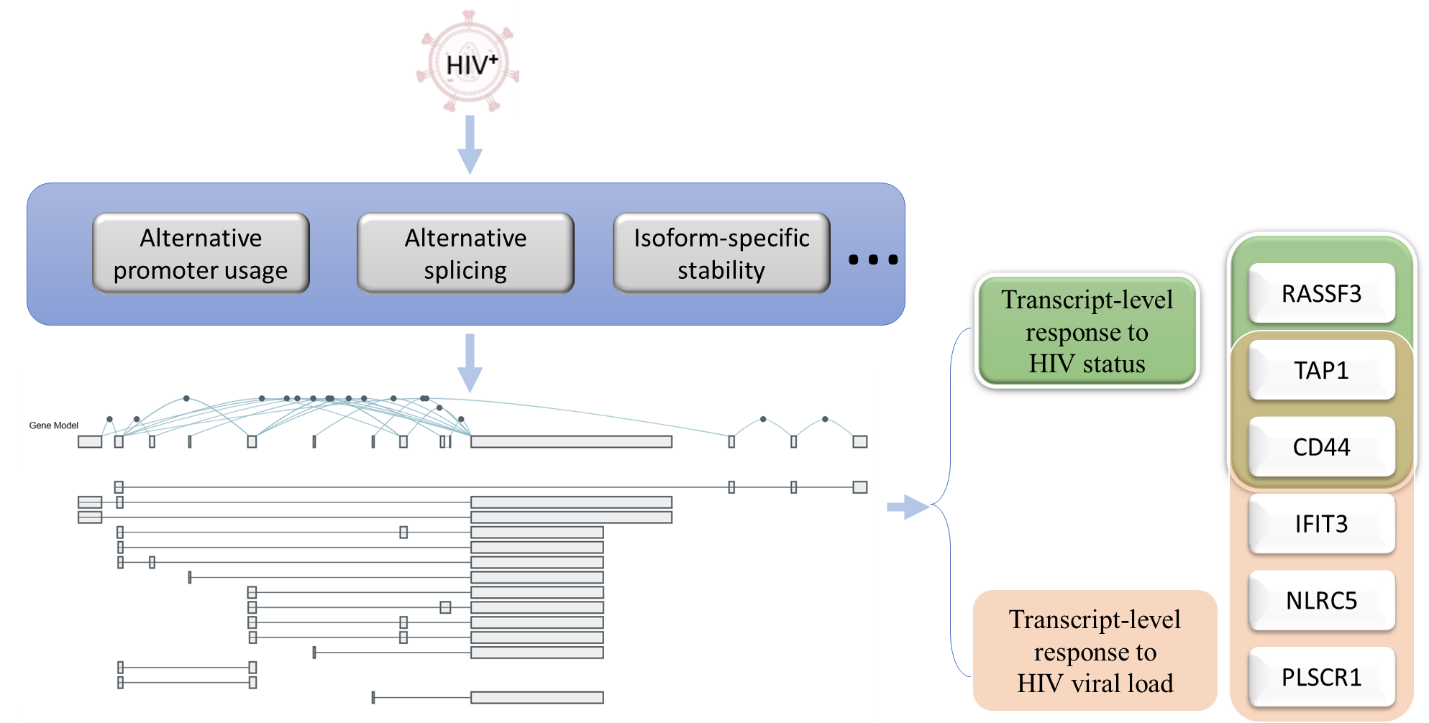


**Supplemental Figure 1**. **Proposed mechanism of nuanced transcriptional regulation linked to viral replication status in PLWH.** This diagram illustrates how HIV status and viral load can trigger post-transcriptional regulatory mechanisms, including alternative promoter usage, alternative splicing, isoform-specific mRNA stability and other possible mechanisms. These processes result in the differential expression of specific isoforms from key immune-related genes. Isoforms of *RASSF3*, *TAP1* and *CD44* were found to be differentially expressed in response to HIV status, while isoforms for *TAP1, CD44, IFIT3*, *NLRC5*, and *PLSCR1* were upregulated only in PLWH with a detectable viral load. This nuanced transcriptional response suggests a sophisticated mechanism for modulating protein function and tailoring immune signaling in response to active viral replication.

1. Gaddis, N. *et al.* Multi-trait genome-wide association study of opioid addiction: OPRM1 and beyond. *Sci Rep* **12**, 16873 (2022).

2. Erly, S., Campos, L., Buskin, S. & Reuer, J. Evaluating surveillance definitions of HIV viral suppression 2015-2019: Which definition best detected barriers to care? *J Public Health Res* **12**, 22799036231182031 (2023).

3. Kim, D., Paggi, J.M., Park, C., Bennett, C. & Salzberg, S.L. Graph-based genome alignment and genotyping with HISAT2 and HISAT-genotype. *Nat Biotechnol* **37**, 907-915 (2019).

4. Patro, R., Duggal, G., Love, M.I., Irizarry, R.A. & Kingsford, C. Salmon provides fast and bias-aware quantification of transcript expression. *Nat Methods* **14**, 417-419 (2017).

5. Soneson, C., Love, M.I. & Robinson, M.D. Differential analyses for RNA-seq: transcript-level estimates improve gene-level inferences. *F1000Res* **4**, 1521 (2015).

6. Andrews, S. FastQC: A quality control tool for high throughput sequence data [Online]. (2010).

7. Li, H. *et al.* The Sequence Alignment/Map format and SAMtools. *Bioinformatics* **25**, 2078-9 (2009).

8. Newman, A.M. *et al.* Determining cell type abundance and expression from bulk tissues with digital cytometry. *Nat Biotechnol* **37**, 773-782 (2019).

9. Newman, A.M. *et al.* Robust enumeration of cell subsets from tissue expression profiles. *Nat Methods* **12**, 453-7 (2015).

10. Shu, C. *et al.* Epigenome-wide association scan identifies methylation sites associated with HIV infection. *Epigenomics* **12**, 1917-1927 (2020).

11. Shu, C. *et al.* DNA methylation mediates the effect of cocaine use on HIV severity. *Clin Epigenetics* **12**, 140 (2020).

12. Shiau, S. *et al.* Distinct epigenetic profiles in children with perinatally-acquired HIV on antiretroviral therapy. *Sci Rep* **9**, 10495 (2019).

13. Zhang, X. *et al.* Epigenome-wide differential DNA methylation between HIV-infected and uninfected individuals. *Epigenetics* **11**, 750-760 (2016).

14. Zhang, X. *et al.* DNA methylation signatures of illicit drug injection and hepatitis C are associated with HIV frailty. *Nat Commun* **8**, 2243 (2017).

15. Love, M.I., Huber, W. & Anders, S. Moderated estimation of fold change and dispersion for RNA-seq data with DESeq2. *Genome Biol* **15**, 550 (2014).

16. Zhu, A., Ibrahim, J.G. & Love, M.I. Heavy-tailed prior distributions for sequence count data: removing the noise and preserving large differences. *Bioinformatics* **35**, 2084-2092 (2019).

17. Anders, S., Reyes, A. & Huber, W. Detecting differential usage of exons from RNA-seq data. *Genome Res* **22**, 2008-17 (2012).

18. Subramanian, A. *et al.* Gene set enrichment analysis: a knowledge-based approach for interpreting genome-wide expression profiles. *Proc Natl Acad Sci U S A* **102**, 15545-50 (2005).

19. Stratford, J.K. *et al.* Identifying compounds to treat opiate use disorder by leveraging multi-omic data integration and multiple drug repurposing databases. *medRxiv* (2024).
